# Testing Cross-Cultural Generalizability of the Task and Ego Orientation in Sport Questionnaire across American and Chinese Samples

**DOI:** 10.1371/journal.pone.0158953

**Published:** 2016-07-11

**Authors:** Cecilia Ma, Eva Monsma

**Affiliations:** 1 Department of Applied Social Sciences, The Hong Kong Polytechnic University, Hunghom, Hong Kong; 2 Department of Physical Education and Athletic Training, University of South Carolina, Columbia, South Carolina, United States of America; IRCCS Istituto Auxologico Italiano, ITALY

## Abstract

This paper examines the factor structure and measurement invariance of the Task and Ego Orientation in Sport Questionnaire (TEOSQ) across American and Chinese samples. Results based on the mean and covariance structure analyses supported configural invariance, metric invariance and scalar invariance across groups. Latent means analyses revealed that American sample had significantly higher mean scores on task and ego orientations than the Chinese sample. The findings suggest that the TEOSQ is a valid and reliable instrument in assessing achievement motivation across these two diverse populations.

## Introduction

Achievement goal theory is one of the most prominent theories of motivation [1]. It provides insight for understanding how individuals interpret, feel, and act when they engage in achievement-related activities [2, 3]. According to Nicholls [4], task-oriented individuals possess undifferentiated conceptions of ability, focus on their own performance, and define their success based on their effort and mastery of the skill. Ego-oriented individuals hold differentiated conceptions of ability, define success by outperforming others with minimum effort, and use norm-referenced information to judge their competence.

Goal theorists contend that people with diverse cultural backgrounds might ascribe different meanings to the definition of success and failure, as well as the conception of ability and attribution beliefs of success and failure, thereby contributing to variations on the perceptions of goal attainment [4, 5]. Researchers have highlighted the role of culture in affecting motivational processes in performing achievement tasks [6, 7]. Supporting evidence for the cultural variation in the perception of achievement goal has been demonstrated by researchers who used the Task and Ego Orientation in Sport Questionnaire (TEOSQ) [8] to examine the generalizability of dispositional goal orientation participants from China [9, 10], Greece [11], Japan [12], Korea[13], and the United Kingdom [14]. In general, the two factor structure of the TEOSQ was supported across diverse populations.

Despite the popularity use of the TEOSQ, it is not clear whether the two goal orientations operate equivalently between Western and Eastern cultures. Previous work has used exploratory factor analysis, which is a data reduction method [15] to assess the factor structure of the TEOSQ and compare the goal orientations between American and Chinese samples. Cross-cultural comparison is only meaningful when the invariance assumption is supported [16]. To date, only one study examined the measurement invariance of the TEOSQ between eastern and western contexts. Li and colleagues [17] examined the measurement invariance and latent mean differences in the two goal orientations among samples from the United States, Taiwan and Thailand. The factorial invariance of the TEOSQ, in terms of configural, metric and scalar measurement, was supported among groups. However, when testing the differences of the latent means, samples from the United States scored significantly higher in both goal orientations as compared with their Taiwan and Thailand counterparts. To further our understanding of the cross-cultural applicability of the achievement goal theories, measurement invariance of the TEOSQ is essential for comparing the true differences in task and ego orientations across groups [16]. The present study extends the literature by testing the cross-cultural measurement equivalence of the TEOSQ among students in Hong Kong and the United States.

Researchers noted that the concept of goal orientation may be different between Western and Eastern societies [10, 18]. Under the influence of Confucianism, physical activity is not typically relevant to promoting mental and social development in Chinese societies [19]. Facing cultural emphasis on academic achievement and a highly competitive education system, Chinese children are encouraged to study at the expense of physical activity [20]. Researchers found decreased interest and participation in physical activities with age as students find it necessary to prepare for upcoming school and public examinations [21]. The emphasis on intellectual development at the expense of physical activity might affect how Chinese people perceive and respond to different physical demands [22].

Physical inactivity is one of the major health risk behaviors in college students [23]. Perhaps, Chinese college students might be less motivated in both dimensions of goal orientation compared to their American counterparts in physical activity domain. This has been supported in the field of academic performance [24]. To date, few studies have considered how achievement goal orientation differs across cultures. Given the global prevalence of physical inactivity, a psychometrically sound instrument will facilitate a meaningful cross-cultural comparison for testing the applicability of the theory in different cultural groups.

Therefore, the purpose of the study is to examine the factorial structure of the TEOSQ between two different cultural groups, American and Chinese samples. It tests the measurement invariance of the TEOSQ and its latent mean differences in two goal orientations across these samples. The following research questions were addressed in the current study:

1. Does the TEOSQ show measurement equivalent across American and Chinese college students in terms of the two-factor structure, loading of item and intercepts?
2. Are there any latent mean differences in the two goal orientations (i.e., task and ego) if the TEOSQ demonstrated invariance of factor loadings and intercepts across the two groups?

It was hypothesized that the two goal orientations will be invariant across two groups. American students will report higher latent means of both goal orientations compared to their Chinese counterparts.

## Materials and Methods

### Participants

The sample included 627 college students from the United State (n = 318) and Hong Kong (n = 309). In the American sample, there were 153 males (48%, mean age = 20.7 years, SD = 2.95) and 165 females (52%, mean age = 20.6 years, SD = 3.33). There were 153 males (50%, mean age = 21.0 years, SD = 1.74) and 156 females (50%, mean age = 21.4 years, SD = 3.01) in the Chinese sample. The ethnic distribution was diverse in American samples (17% African American, 3% Asian American, 10% Native Hawaiian, 60% White, and 10% other groups) while the Chinese sample consisted of both Hong Kong and Mainland Chinese (70% Hong Kong Chinese; 30% Mainland Chinese). Participants were recruited from two public urban university campuses, one from the southeastern United States and one from Hong Kong. Both universities provide similar on-campus physical activities facilities (i.e., two main sport centers with open recreational areas), which are oriented towards physically active leisure activities. In the American sample, 22% were freshmen, 25%, sophomores, 15% juniors, 26%, seniors, and 12% graduate students. On the other hand, the Chinese sample constituted approximately 44% freshmen, 35% sophomores, 14% juniors, and 7% graduate students. The university in Hong Kong was a three-year institution. Overall, the majority of participants in both cultural groups were full-time students (Americans: 96% & Chinese: 100%). Ethics approval of the research protocol was obtained from University of South Carolina and The Hong Kong Polytechnic University prior to initiating the study. Furthermore, all participants were asked to read and sign a consent form before completing the questionnaire.

### Measures

The Task and Ego Orientation in Sport Questionnaire (TEOSQ) [8] was used to measure students’ goal orientations. This scale consists of 7 task-related and 6 ego-related items reflecting the definitions of success in sport contexts. The items are prefaced with the heading “*I feel most successful in this class when*…” Students rated each item on a 5-point Likert-type scale ranging from *strongly disagree* (1) to *strongly agree*. In the present study, the 13-item TEOSQ scale showed good reliability in both samples (α = .82-.84 for Chinese sample; α = .79-.91 for American sample, S1 Table).

#### Translation procedure

A back-translation procedure was used to measure the meaning equivalence of the instrument [25]. The original questionnaires were developed in English and were translated into Chinese by the author who is fluent in both English and Chinese. Without referring to the original scale, backward translation was done by two Chinese translators, who are bilingual and fluent in English and Chinese. Then an independent scholar compared the backward translation versions with the original versions to ensure the content of both versions was the same. No deviation in meaning was found.

### Data Analysis

We tested the factorial invariance of the TEOSQ in terms of: a) configural invariance, b) factor loadings, c) intercepts of the measured variable, and d) latent factor means. To evaluate the model fit, several fit statistics were used, including chi-square goodness-of-fit test (χ^2^), comparative fit index (CFI), nonnormed fit index (NNFI), standardized root-mean-square residual (SRMR), expected cross-validation index (ECVI) and root-mean-square error of approximation (RMSEA). For CFI and NNFI, the values of .90 indicate an acceptable fit and values .95 or greater suggest a satisfactory fit to the data [26–27]. The values of RMSEA below .08 reflect an acceptable fit [28]. For the SRMR, values below .08 indicate acceptable fit [27]. For the values of ECVI, a smaller value indicates the better fit of the model [29]. Modification index (MI) is used to locate the sources of model misspecification [29]. To assess the results of measurement invariance between two nested models, changes in CFI and chi-square difference values were used [30]. The insignificant chi-square difference test (Δχ^2^) and the value of ΔCFI less than or equal to .01 suggests that the null hypothesis of invariance should be retained [30]. Following the general practice in invariance testing [16, 30], a two-factor model was tested in each group, followed by a series of increasingly restrictive constraint to test the invariance of factor structure (configural invariance), the invariance of factor loading (metric invariance) and the invariance of intercept (scalar invariance). The invariance of latent means was tested once the factor loadings and intercepts were invariant across groups.

To attain statistical identification purpose, one item from each factor was fixed to a value of 1.0. All analyses were conducted by using the covariance and mean matrices via LISREL 8.80 [31]. Assumptions of normality (i.e., the multivariate kurtosis was below 3.0, the univariate skewness and kurtosis values were lower than 2 and 7, respectively) [32–33] and homoscedasticity (correlations are below .70; Tolerance: above .10; VIF: below 5.0) were supported, maximum likelihood estimation (ML) was used. Five percent of the data were missing in both samples, thus, the listwise deletion method was used for handling the missing data.

## Results

S2 Table shows the goodness-of-fit indexes of all models. Before performing the invariance test, the two-factor structure of TEOSQ was examined in each sample (Model 1: American sample; Model 2: Chinese sample). For the American sample, we adopted the suggestion from an anonymous reviewer by excluding Asian American in the study. This model reached an acceptable level. High MI values are found in a pair of error covariance (Item7 and Item 13: MI = 40.73). This parameter is freely estimated as these items belong to the same construct. This parameter is freely estimated in the subsequent models (Model 1a). This model fits the data better (Model 1a: χ^2^_(63)_ = 156.49, *p* < .01; CFI = .96; NNFI = .95; RMSEA = .06; SRMR = .05; EVCI = .65). Similar to the American sample, the two-factor model of the TEOSQ reached an acceptable level. Same pair of error covariance (Item7 and Item 13: MI = 43.35) is suggested to be freely estimated in the revised model (Model 2a). This model showed satisfactory fit indices (Model 2a: χ^2^_(63)_ = 159.20, *p* < .01; CFI = .96; NNFI = .95; RMSEA = .07; SRMR = .06; EVCI = .73). The factor loadings of the two-factor TEOSQ model in both samples are shown in figures (S1 and S2 Figs).

In testing for the invariance of the TEOSQ, we first estimated whether an equal number of factors (i.e., configural invariance) across cultural groups. In this model, no constraints are imposed in both groups (Model 3). This model offers good fit with the data, suggesting the factor structure is invariant across groups (Model 3: χ^2^_(126)_ = 315.69, *p* < .01; CFI = .96; NNFI = .95; RMSEA = .07; SRMR = .06; EVCI = .73).

We then tested metric invariance by constraining all factor loadings invariant (Model 4). Compared with the baseline model (Model 3), no change in ΔCFI and Δχ^2^, indicating metric invariance is supported across cultural groups [30]. Given that the invariance of configural and metric measurement are supported, the scalar measurement invariance is tested to examine the cultural heterogeneity across groups in Model 5 [16, 30]. The invariance of intercepts was supported with insignificant changes in CFI (ΔCFI = .01), despite the significance of Δχ^2^ = 81.77, Δdf = 13, *p* < .01, Model 5). We then tested the invariance of latent means across groups (Model 6). The assumption of the invariant of latent mean was not supported (i.e., ΔCFI = .34). By comparing the latent means, American groups scored significantly higher than Chinese group in task (mean = .78, *t* = 18.09, *SE* = .04, *p* < .01) and ego orientations (mean = .69, *t* = 8.72, *SE* = .08, *p* < .01).

## Discussion

The present study examined the factor structure and invariance of the TEOSQ across American and Chinese samples. Results of configural invariance suggest that the two-factor structure was supported in both cultural groups indicating that the two goal orientations are the same in two samples. The overall TEOSQ structure appears to be consistent across cultural groups. It supports the applicability of the two-goal orientations in non-western contexts.

The findings of metric invariance indicating the strength of the relationships of the items with the corresponding factors are equal. Similar results were found in the test of scalar measurement invariance. These results imply that the TEOSQ operates equivalently in both American and Chinese college students thus supporting its further use in Chinese culture particularly for answering physical activity research questions pertaining to emerging health related outcomes that are similar to those in Western societies.

When testing the differences in latent mean scores, the American sample reported higher mean scores in both goal orientations than did the Chinese sample. Indeed, American respondents scored higher in both goal orientations than their Chinese counterparts, and these results are consistent with findings in the context of physical [10, 17] and academic [24] domains. There are two possible explanations for this finding. First, facing cultural emphasis on academic achievement and a highly competitive education system, Chinese students consider the need for “study” as a barrier for engaging in physical activity [34]. *This barrier* highlights cultural differences in physical activity motivation, as this has never been demonstrated in studies which were conducted in Western countries. Second, Chinese students have been socialized to be modest and humble [35] and seem more likely to respond in middle response as shown in prior work [10, 36]. The findings of the present study extend the literature by showing the role of culture on influencing the latent means of the two goal orientations among samples in American and Chinese contexts.

Understanding the goal orientation can be relevant to health practitioners and professionals to develop appropriate program to motivate individual’s participation in physical activity. Aligned with increasing evidence that physical activity supports executive function, and benefits learning and retention [37], promoting physical activity in conjunction with learning and studying habits should be advocated. In addition to challenging studying behaviors confined by Confucianism, future investigations of achievement motivation among Asian populations should consider expanding the task and ego constructs represented by the TEOSQ to include social orientation in the study of cross-cultural achievement goal research. For example, how the presence of a harmonious relationship with others will influence individuals ‘involvement in physical activity differently between western and eastern cultural groups. The emphasis on “maintaining social harmony” in collectivistic culture might affect individual achievement-related cognitions and behaviors as demonstrated in academic research [38].

The findings of the present study provide strong evidence for the appropriateness of the Chinese-language version of the TEOSQ in non-western context. It demonstrates the cross-cultural generalizability of the two-factor structure of the TEOSQ in Chinese contexts. It provides meaningful latent mean differences across the two cultural groups. This extends the traditional methods, such as multivariate analysis of variance by comparing the differences in group means. Several limitations need to be highlighted. In the present study, Hong Kong students were recruited. The findings might not generalize to Chinese sample in China, Taiwan and Singapore. Future research should include samples from other Asian countries and age group in order to provide additional data to support the validity of the TEOSQ. It might also be interesting to examine measurement invariance across gender in other racial and ethnic groups. Spence [39] noted the paucity of research in addressing the possible moderators, such as gender, ethnicity when studying the behavioral intention in physical contexts. Given the evidence of two goal orientations of the instrument, future study might evaluate the relationship between goal orientations and other psychological well-being and behavior in other physical contexts. Lastly, future research might use a qualitative approach to elicit how global and contextual motivations are conceptualized in different cultural backgrounds.

## Supporting Information

S1 Fig Factor structure and completely standardized factor loading on goal orientation in the American sample. (TIF)

S2 Fig Factor structure and completely standardized factor loading on goal orientation in the Chinese sample. (TIF)

S1 Table Descriptive statistics, internal consistency for American and Chinese samples. (TIF)

S2 Table Summary of Goodness of Fit for dispositional goals. (TIF)
