# Supplementary figures and images for: Testing Cross-Cultural Generalizability of the Task and Ego Orientation in Sport Questionnaire across American and Chinese Samples
Source: PLoS One. 2016 Jul 11;11(7):e0158953. doi: 10.1371/journal.pone.0158953 (PMC4939965; doi:10.1371/journal.pone.0158953)

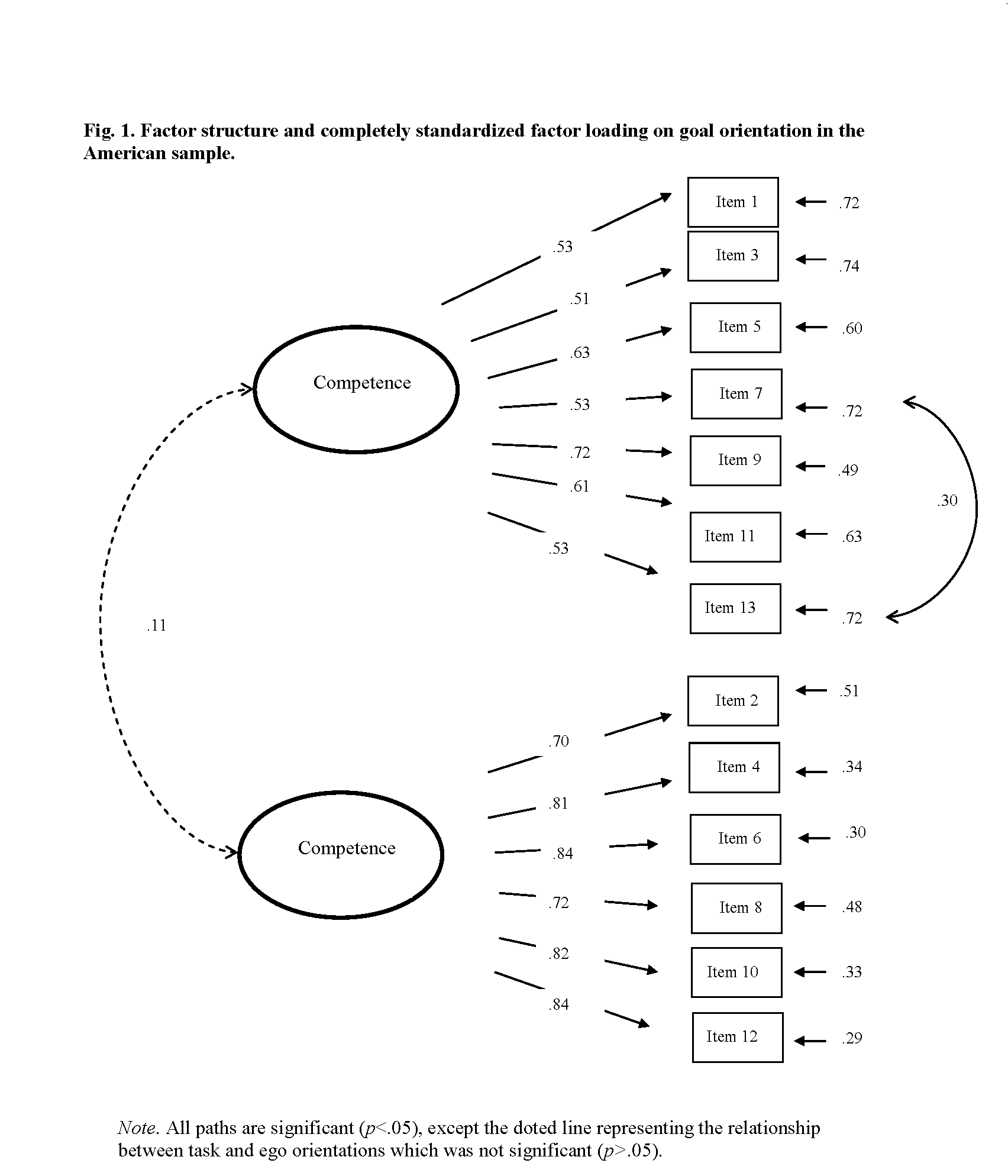

Supplement: S1 Fig — (TIF) [file pone.0158953.s001.tif]

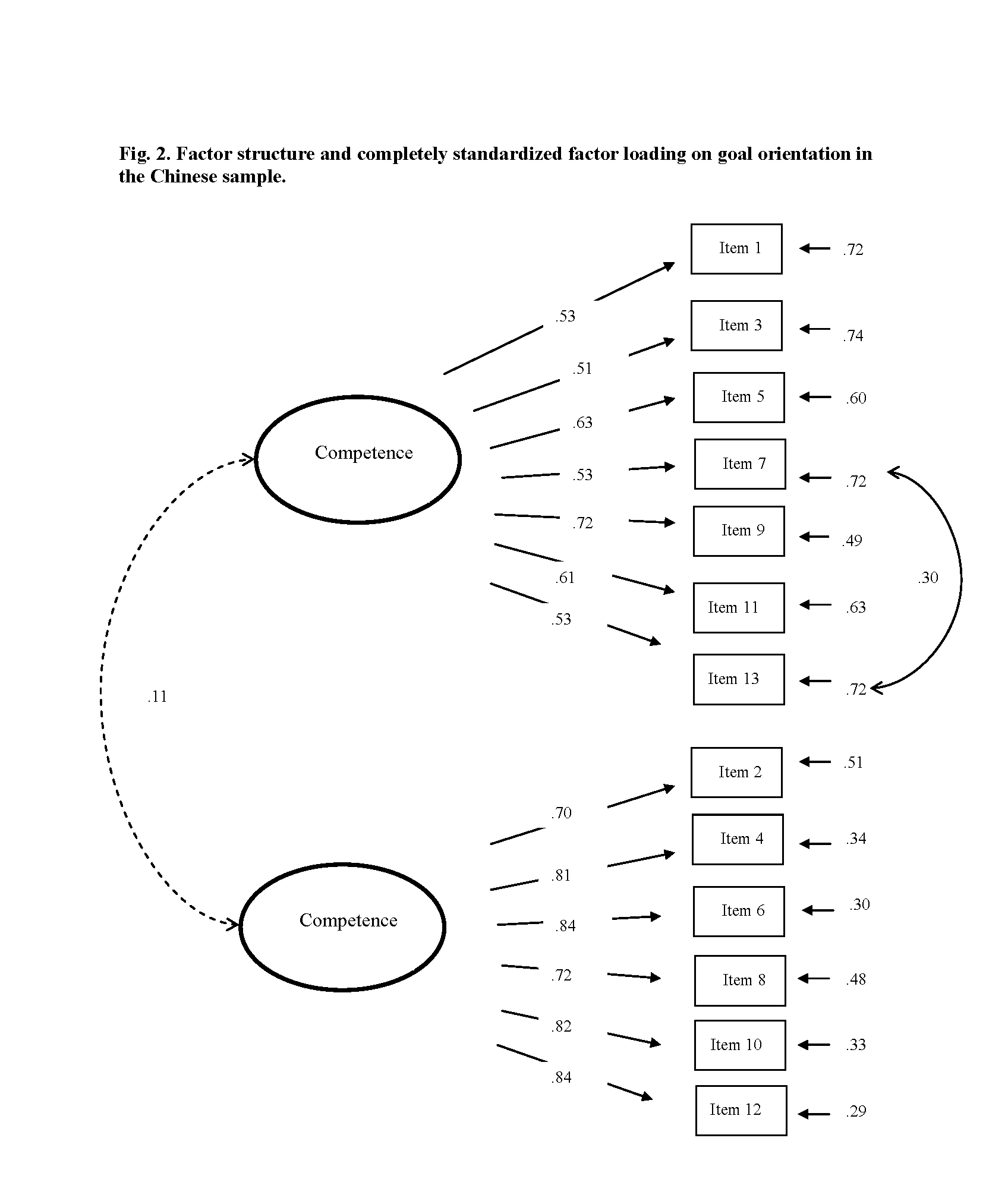

Supplement: S2 Fig — (TIF) [file pone.0158953.s002.tif]

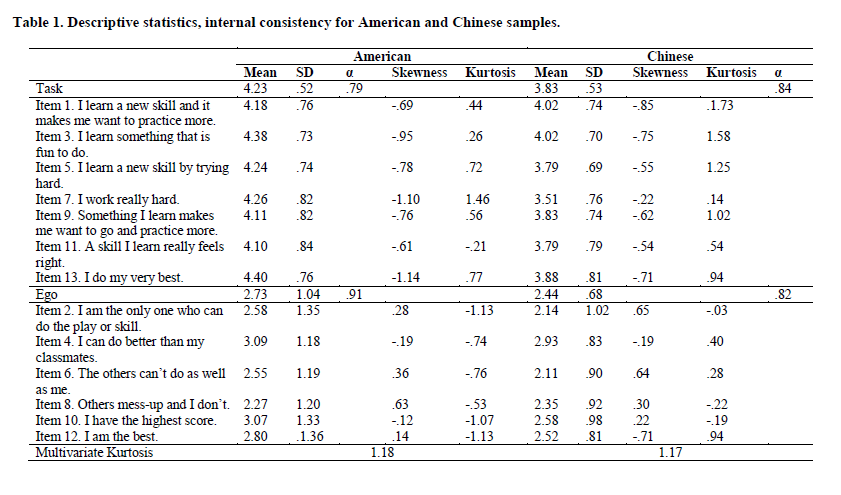

Supplement: S1 Table — (TIF) [file pone.0158953.s003.tif]

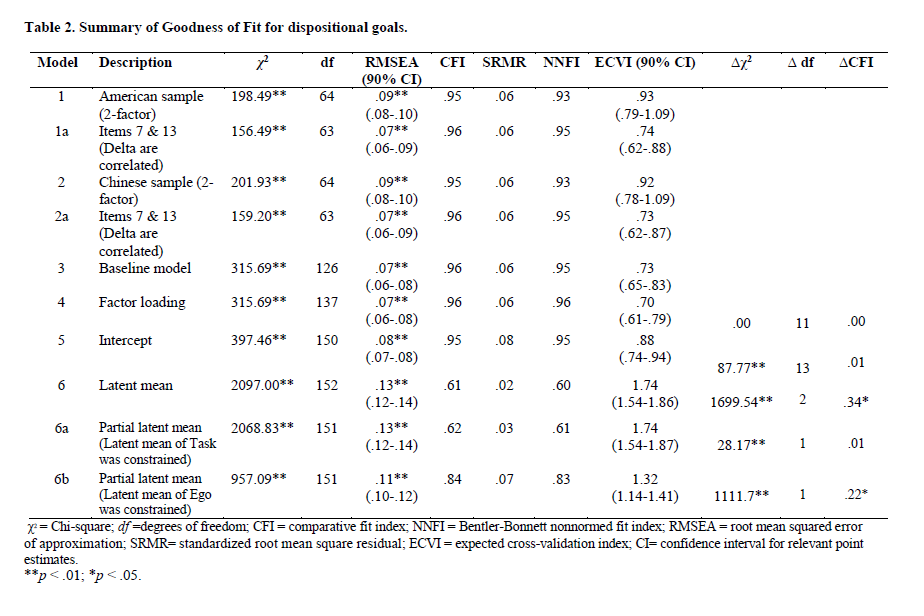

Supplement: S2 Table — (TIF) [file pone.0158953.s004.tif]
